# Methodological approaches to the study of cancer risk in the vicinity of pollution sources: the experience of a population-based case–control study of childhood cancer

**DOI:** 10.1186/s12942-019-0176-x

**Published:** 2019-05-28

**Authors:** Javier García-Pérez, Diana Gómez-Barroso, Ibon Tamayo-Uria, Rebeca Ramis

**Affiliations:** 1 Cancer and Environmental Epidemiology Unit, Department of Epidemiology of Chronic Diseases, National Center for Epidemiology, Carlos III Institute of Health, Avda. Monforte de Lemos, 5, 28029 Madrid, Spain; 2 Consortium for Biomedical Research in Epidemiology and Public Health (CIBER Epidemiología y Salud Pública - CIBERESP), Madrid, Spain; 3 National Center for Epidemiology, Carlos III Institute of Health, Madrid, Spain; 4 Division of Immunology and Immunotherapy, Cima Universidad de Navarra and “Instituto de Investigación Sanitaria de Navarra (IdISNA)”, Pamplona, Spain

**Keywords:** Cancer risk, Childhood cancer, Methodology, Industrial pollution, Urban pollution, Case–control study

## Abstract

**Background:**

Environmental exposures are related to the risk of some types of cancer, and children are the most vulnerable group of people. This study seeks to present the methodological approaches used in the papers of our group about risk of childhood cancers in the vicinity of pollution sources (industrial and urban sites). A population-based case–control study of incident childhood cancers in Spain and their relationship with residential proximity to industrial and urban areas was designed. Two methodological approaches using mixed multiple unconditional logistic regression models to estimate odds ratios (ORs) and 95% confidence intervals (95% CIs) were developed: (a) “*near vs. far*” analysis, where possible excess risks of cancers in children living near (“near”) versus those living far (“far”) from industrial and urban areas were assessed; and (b) “*risk gradient*” analysis, where the risk gradient in the vicinity of industries was assessed. For each one of the two approaches, three strategies of analysis were implemented: “*joint*”, “*stratified*”, and “*individualized*” analysis. Incident cases were obtained from the Spanish Registry of Childhood Cancer (between 1996 and 2011).

**Results:**

Applying this methodology, associations between proximity (≤ 2 km) to specific industrial and urban zones and risk (OR; 95% CI) of leukemias (1.31; 1.04–1.65 for industrial areas, and 1.28; 1.00–1.53 for urban areas), neuroblastoma (2.12; 1.18–3.83 for both industrial and urban areas), and renal (2.02; 1.16–3.52 for industrial areas) and bone (4.02; 1.73–9.34 for urban areas) tumors have been suggested.

**Conclusions:**

The two methodological approaches were used as a very useful and flexible tool to analyze the excess risk of childhood cancers in the vicinity of industrial and urban areas, which can be extrapolated and generalized to other cancers and chronic diseases, and adapted to other types of pollution sources.

## Background

Environmental exposures are related to the risk of some types of cancer [1], and children are the most vulnerable group of people because they are far more sensitive than adults to toxic chemicals in the environment [2, 3]. Moreover, the causes of many childhood cancers are largely unknown, so it is necessary epidemiologic research as a tool for identifying associations between proximity to environmental exposures and the frequency of these cancers. In this sense, the biggest population-based case–control study of incident childhood cancer in Spain has been carried out by our group with the purpose of analyzing the risk of various types of cancer in the proximity of environmental exposures (industrial installations, urban areas, road traffic, and agricultural crops) [4–12].

This paper seeks: (a) to present the several methodological approaches used in our study, summarizing the main results; and, (b) to describe our experience studying the risk of childhood cancers in the vicinity of some of the pollution point sources, principally industrial and urban sites, with the purpose of establishing some guidelines and encouraging other researchers to apply these methodological tools in their environment-epidemiologic studies, using the publicly-available data from the Pollutant Release and Transfer Registers (PRTRs).

## Results

Spanish industrial installations included in the European PRTR (E-PRTR) were taken into account in the paper. A list of industrial groups, together with their E-PRTR categories, and number of industrial installations and amounts (in kg) released by these industrial plants in 2009, by groups of carcinogens [according to the International Agency for Research on Cancer (IARC)] and groups of toxic substances, are shown in Table 1. A list including the specific pollutants released to both air and water, by category of industrial groups, are described in detail in Table 2.

**Table 1:** Industrial groups and amounts (in kg) released by facilities in 2009, by groups of carcinogens and toxic substances

Industrial group (no. industries)	E-PRTR category	IARC groups^a^ (amounts in kg)			Groups of toxic substances^b^ (amounts in kg)								
		Group 1	Group 2A	Group 2B	Metals	Pesticides	PACs	Non-HPCs	Plasticizers	POPs	VOCs	Solvents	Other
Combustion installations (42)	1.c	1,311,336	275	0	5029	0	548	0	0	548	333,457	1676	1,307,481
Refineries and coke ovens (4)	1.a, 1.d	443,462	206	22	18,556	0	315	0	0	315	2,810,616	5177	422,599
Production and processing of metals (119)	2.a, 2.b, 2.c.i, 2.c.ii, 2.d, 2.e	1,172,280	12,911	34	160,275	35	2212	2	0	2223	830,065	33,221	1,132,947
Galvanization (19)	2.c.iii	4389	95	0	1085	0	0.02	0	0	0.02	719	0	4367
Surface treatment of metals and plastic (197)	2.f	68,828	580	206	10,290	87	12	0	200	99	2,898,336	1490	63,145
Mining industry (39)	3.a, 3.b	1,246,894	0	0	0	0	0	0	0	0	48,613	0	1,246,894
Cement and lime (33)	3.c, 3.d	1,429,626	331	1085	1777	0	415	0	560	334	304,099	6405	1,422,443
Glass and mineral fibers (20)	3.e, 3.f	419,668	1715	92	3980	0	5	0.001	0	5	2,506,147	870	417,528
Ceramic (86)	3.g	560,042	262	2	2543	0	0.02	0	0	0.02	109,861	410	558,235
Organic chemical industry (106)	4.a	375,168	432	19,137	3221	0.1	472	2042	0.2	465	2,758,750	22,757	308,124
Inorganic chemical industry (46)	4.b	56,575	77	18	1249	3	0.1	0	0	4	16,041	19	55,957
Fertilizers (10)	4.c	23,512	0	2	537	0	0	0	0	0	59	0	23,512
Biocides (12)	4.d	4601	81	0.2	21	0	0	0	0	0	2860	81	4601
Pharmaceutical products (41)	4.e	2561	314,238	91,882	436	0	0.01	0	0	0.01	3,252,059	406,243	2480
Explosives and pyrotechnics (9)	4.f	111	374	0	395	0	0	0	0	0	454	0	101
Hazardous waste (60)	5.a, 5.b	29,578	417	70	1976	0.4	95	0	0	96	54,009	259	28,718
Non-hazardous waste (86)	5.c, 5.d	18,551	331	64	8490	54	0.3	17	6	33	138,044	357	16,210
Disposal or recycling of animal waste (18)	5.e	23,136	0	0	2	0	0.8	0	0	0.8	5397	0	23,135
Urban waste-water treatment plants (53)	5.f, 5.g	10,834	1432	80	43,128	174	49	554	0	48	173,123	107	172
Paper and wood production (63)	6.a, 6.b, 6.c	547,721	146	1	1863	11	0.4	0	0.02	11	1,372,034	4494	542,628
Pre-treatment or dyeing of textiles (9)	9.a	2278	0	0	60	0	0	0	0	0	6238	0	2274
Tanning of hides and skins (2)	9.b	18	0	0	18	0	0	0	0	0	139	0	0
Food and beverage sector (145)	8.a, 8.b, 8.c	244,617	1	0.01	377	0	0.04	0	0	0.01	593,932	0.3	244,539
Surface treatment using organic solvents (50)	9.c	63,019	193	203	2964	67	0.01	0	0	67	8,837,821	1812	62,608
Production of carbon or electro-graphite (2)	9.d	18,917	0	0	0	0	37	0	0	37	8500	0	18,880
Total (1271)		8,077,722	334,095	112,899	268,272	431	4162	2615	766	4287	27,061,375	485,378	7,909,578

*OR* odds ratio, *95% CI* 95% confidence interval

^a^IARC carcinogenic classification: Group 1: carcinogens to humans (arsenic and compounds, cadmium and compounds, chromium and compounds, nickel and compounds, lindane, dioxins + furans, polychlorinated biphenyls, trichloroethylene, vinyl chloride, benzene, ethylene oxide, polycyclic aromatic hydrocarbons, particulate matter (PM_10_), total suspended particulate matter, and benzo(a)pyrene); Group 2A: probably carcinogenic to humans (lead and compounds, dichloromethane, tetrachloroethylene, DDT, and hexabromobiphenyl); Group 2B: possibly carcinogenic to humans (chlordane, 1,2-dichloroethane, dichloromethane, heptachlor, hexachlorobenzene, 1,2,3,4,5,6-hexachlorocyclohexane, lindane, mirex, pentachlorophenol, tetrachloromethane, trichloromethane, ethyl benzene, naphthalene, di-(2-ethyl hexyl) phthalate, cobalt and compounds, benzo(b)fluoranthene, benzo(k)fluoranthene, and indeno(1,2,3-cd)pyrene)

^b^Metals (arsenic and compounds, cadmium and compounds, chromium and compounds, copper and compounds, mercury and compounds, nickel and compounds, lead and compounds, zinc and compounds, thallium, antimony, cobalt, manganese, and vanadium); Pesticides (alachlor, aldrin, atrazine, chlordane, chlorfenvinphos, chlorpyrifos, DDT, dieldrin, diuron, endosulfan, endrin, heptachlor, lindane, mirex, pentachlorobenzene, pentachlorophenol, simazine, isoproturon, organotin compounds, tributyltin and compounds, triphenyltin and compounds, trifluralin, and isodrin); PACs: Polycyclic aromatic chemicals (anthracene, polycyclic aromatic hydrocarbons, fluoranthene, benzo(g,h,i)perylene, benzo(a)pyrene, benzo(b)fluoranthene, benzo(k)fluoranthene, and indeno(1,2,3-cd)pyrene); Non-HPCs: Non-halogenated phenolic chemicals (nonylphenol and nonylphenol ethoxylates, and octylphenols and octylphenol ethoxylates); Plasticizers (di-(2-ethyl hexyl) phthalate); POPs: Persistent organic pollutants (aldrin, chlordane, DDT, dieldrin, endosulfan, endrin, heptachlor, hexachlorobenzene, 1,2,3,4,5,6-hexachlorocyclohexane, lindane, mirex, dioxins + furans, pentachlorobenzene, polychlorinated biphenyls, brominated diphenylethers, organotin compounds, polycyclic aromatic hydrocarbons, hexabromobiphenyl, benzo(a)pyrene, benzo(b)fluoranthene, and benzo(k)fluoranthene); VOCs: Volatile organic compounds (non-methane volatile organic compounds, 1,2-dichloroethane, dichloromethane, hexachlorobutadiene, tetrachloroethylene, trichlorobenzenes, 1,1,1-trichloroethane, trichloroethylene, trichloromethane, vinyl chloride, benzene, ethyl benzene, ethylene oxide, and naphthalene); Solvents (1,2-dichloroethane, dichloromethane, tetrachloroethylene, trichlorobenzenes, 1,1,1-trichloroethane, trichloroethylene, trichloromethane, benzene, ethyl benzene, toluene, and xylenes); Other (tetrachloromethane, particulate matter (PM_10_), and total suspended particulate matter)

**Table 2:** Specific pollutants released to both air and water, by industrial groups

Industrial group	Pollutants released by industrial groups	
	Air	Water
Combustion installations	NMVOC^a^, arsenic, cadmium, chromium, copper, mercury, nickel, lead, zinc, dioxins + furans, trichloroethylene, benzene, PAHs^b^, PM_10_^c^, TSP^d^, manganese, vanadium	Arsenic, cadmium, chromium, copper, mercury, nickel, lead, zinc, dioxins + furans, PAHs^b^, toluene, fluoranthene, benzo(g,h,i)perylene
Refineries and coke ovens	NMVOC^a^, arsenic, cadmium, chromium, copper, mercury, nickel, lead, zinc, dioxins + furans, polychlorinated biphenyls, 1,1,1-trichloroethane, trichloromethane, anthracene, benzene, naphthalene, PAHs^b^, PM_10_^c^, TSP^d^, antimony, cobalt, manganese, vanadium, ethyl benzene	Arsenic, cadmium, chromium, copper, mercury, nickel, lead, zinc, dioxins + furans, benzene, PAHs^b^, toluene, xylenes, fluoranthene, benzo(g,h,i)perylene
Production and processing of metals	NMVOC^a^, arsenic, cadmium, chromium, copper, mercury, nickel, lead, zinc, hexachlorobenzene, lindane, dioxins + furans, polychlorinated biphenyls, anthracene, benzene, naphthalene, PAHs^b^, PM_10_^c^, TSP^d^	Arsenic, cadmium, chromium, copper, mercury, nickel, lead, zinc, pentachlorophenol, anthracene, nonylphenol, naphthalene, organotin compounds, PAHs^b^, octylphenols, fluoranthene, benzo(g,h,i)perylene, benzo(a)pyrene, benzo(b)fluoranthene, benzo(k)fluoranthene, indeno(1,2,3-cd)pyrene
Galvanization	NMVOC^a^, arsenic, cadmium, chromium, copper, nickel, lead, zinc, dioxins + furans, PM_10_^c^, TSP^d^	Arsenic, cadmium, chromium, copper, nickel, lead, zinc, PAHs^b^
Surface treatment of metals and plastic	NMVOC^a^, cadmium, chromium, copper, mercury, nickel, lead, zinc, benzene, dichloromethane, 1,2,3,4,5,6-hexachlorocyclohexane, tetrachloroethylene, trichloroethylene, di-(2-ethyl hexyl) phthalate, PAHs^b^, PM_10_^c^, TSP^d^, manganese, vanadium	Arsenic, cadmium, chromium, copper, mercury, nickel, lead, zinc, anthracene, naphthalene, organotin compounds, di-(2-ethyl hexyl) phthalate, PAHs^b^, fluoranthene, trichloromethane, toluene, benzo(b)fluoranthene, ethyl benzene, xylenes
Mining industry	NMVOC^a^, PM_10_^c^, TSP^d^	
Cement and lime	NMVOC^a^, arsenic, cadmium, chromium, copper, mercury, nickel, lead, zinc, dioxins + furans, polychlorinated biphenyls, anthracene, benzene, naphthalene, di-(2-ethyl hexyl) phthalate, PAHs^b^, PM_10_, TSP^d^, thallium, antimony, cobalt, manganese, vanadium	Copper, zinc
Glass and mineral fibers	NMVOC^a^, arsenic, cadmium, chromium, copper, mercury, nickel, lead, zinc, dioxins + furans, polychlorinated biphenyls, benzene, PAHs^b^, PM_10_^c^, TSP^d^, manganese, vanadium	Arsenic, cadmium, chromium, copper, mercury, nickel, lead, zinc, benzene, ethyl benzene, toluene, xylenes, octylphenols
Ceramic	NMVOC^a^, arsenic, cadmium, chromium, copper, mercury, nickel, lead, zinc, benzene, PAHs^b^, PM_10_^c^, TSP^d^, thallium, antimony, cobalt, manganese, vanadium	Arsenic, cadmium, chromium, copper, mercury, nickel, lead, zinc, trichloromethane, naphthalene
Organic chemical industry	NMVOC^a^, arsenic, cadmium, chromium, copper, mercury, nickel, lead, zinc, 1,2-dichloroethane, dichloromethane, dioxins + furans, tetrachloroethylene, tetrachloromethane, trichloromethane, vinyl chloride, anthracene, benzene, ethylene oxide, naphthalene, PAHs^b^, PM_10_^c^, TSP^d^, antimony, cobalt, manganese, vanadium	Arsenic, cadmium, chromium, copper, mercury, nickel, lead, zinc, aldrin, atrazine, chlordane, chlorfenvinphos, chlorpyrifos, DDT, 1,2-dichloroethane, dichloromethane, dieldrin, endosulfan, endrin, hexachlorobenzene, hexachlorobutadiene, 1,2,3,4,5,6-hexachlorociclohexane, mirex, dioxins + furans, simazine, tetrachloroethylene, trichlorobenzenes, trichloroethylene, trichloromethane, vinyl chloride, anthracene, benzene, brominated diphenylethers, nonylphenol, ethyl benzene, naphthalene, organotin compounds, di-(2-ethyl hexyl) phthalate, PAHs^b^, toluene, tributyltin, xylenes, octylphenols, fluoranthene, isodrin, benzo(b)fluoranthene, indeno(g,h,i)perylene
Inorganic chemical industry	NMVOC^a^, arsenic, cadmium, chromium, copper, mercury, nickel, lead, zinc, dichloromethane, dioxins + furans, tetrachloromethane, trichloromethane, PM_10_^c^, TSP^d^, antimony	Arsenic, cadmium, chromium, copper, mercury, nickel, lead, zinc, hexachlorobenzene, dioxins + furans, trichloromethane, organotin compounds, PAHs^b^, fluoranthene
Fertilizers	NMVOC^a^, zinc, PM_10_^c^, TSP^d^, cobalt	
Biocides	NMVOC^a^, dichloromethane, PM_10_^c^	Copper, zinc, ethyl benzene, xylenes
Pharmaceutical products	NMVOC^a^, arsenic, cadmium, chromium, copper, mercury, nickel, lead, zinc, 1,2-dichloroethane, dichloromethane, tetrachloromethane, trichloromethane, PM_10_^c^, TSP^d^, thallium, antimony, cobalt, manganese, vanadium	Chromium, copper, mercury, lead, zinc, 1,2-dichloroethane, dichloromethane, tetrachloroethylene, tetrachloromethane, trichloroethylene, trichloromethane, benzene, ethyl benzene, toluene, xylenes, naphthalene, PAHs^b^, fluoranthene, benzo(a)pyrene, benzo(b)fluoranthene, benzo(k)fluoranthene
Explosives and pyrotechnics	NMVOC^a^, lead, PM_10_^c^	Arsenic, cadmium, chromium, copper, mercury, nickel, lead, zinc
Hazardous waste	NMVOC^a^, arsenic, cadmium, chromium, copper, mercury, nickel, lead, zinc, hexachlorobenzene, dioxins + furans, tetrachloroethylene, trichloroethylene, benzene, PAHs^b^, PM_10_^c^, TSP^d^, thallium, antimony, cobalt, manganese, vanadium	Arsenic, cadmium, chromium, copper, mercury, nickel, lead, zinc, dichloromethane, benzene, polychlorinated biphenyls, tetrachloroethylene, trichloroethylene, trichloromethane, ethyl benzene, naphthalene, organotin compounds, PAHs^b^, toluene, xylenes
Non-hazardous waste	NMVOC^a^, arsenic, cadmium, chromium, copper, mercury, nickel, lead, zinc, dioxins + furans, dichloromethane, tetrachloroethylene, tetrachloromethane, trichloroethylene, vinyl chloride, PM_10_^c^, TSP^d^, antimony, cobalt, manganese, vanadium	Arsenic, cadmium, chromium, copper, mercury, nickel, lead, zinc, alachlor, aldrin, atrazine, chlordane, chlorfenvinphos, chlorpyrifos, DDT, 1,2-dichloroethane, dichloromethane, dieldrin, diuron, endosulfan, endrin, heptachlor, hexachlorobenzene, hexachlorobutadiene, 1,2,3,4,5,6-hexachlorociclohexane, lindane, mirex, dioxins + furans, pentachlorobenzene, pentachlorophenol, polychlorinated biphenyls, simazine, tetrachloroethylene, trichlorobenzenes, trichloroethylene, trichloromethane, vinyl chloride, anthracene, benzene, brominated diphenylethers, nonylphenol, ethyl benzene, isoproturon, naphthalene, organotin compounds, di-(2-ethyl hexyl) phthalate, PAHs^b^, toluene, tributyltin, triphenyltin, trifluralin, xylenes, octylphenols, fluoranthene, isodrin, hexabromobiphenyl
Disposal or recycling of animal waste	NMVOC^a^, PAHs^b^, dioxins + furans, PAHs^b^, PM_10_^c^, TSP^d^	Zinc, dioxins + furans
Urban waste-water treatment plants	NMVOC^a^, cadmium, chromium, copper, mercury, nickel, lead, dioxins + furans, PM_10_^c^	Arsenic, cadmium, chromium, copper, mercury, nickel, lead, zinc, atrazine, 1,2-dichloroethane, diuron, lindane, pentachlorophenol, simazine, tetrachloroethylene, tetrachloromethane, trichloromethane, anthracene, benzene, nonylphenol, ethyl benzene, isoproturon, naphthalene, organotin compounds, di-(2-ethyl hexyl) phthalate, PAHs^b^, toluene, tributyltin, xylenes, octylphenols, fluoranthene, benzo(g,h,i)perylene, benzo(a)pyrene, benzo(b)fluoranthene, benzo(k)fluoranthene, indeno(1,2,3-cd)pyrene
Paper and wood production	NMVOC^a^, arsenic, cadmium, chromium, copper, mercury, nickel, lead, zinc, di-(2-ethyl hexyl) phthalate, PM_10_^c^, TSP^d^	Arsenic, cadmium, chromium, copper, mercury, nickel, lead, zinc, tetrachloroethylene, trichlorobenzenes, trichloroethylene, trichloromethane, organotin compounds, di-(2-ethyl hexyl) phthalate, PAHs^b^, toluene
Pre-treatment or dyeing of textiles	NMVOC^a^, PM_10_^c^	Chromium, copper, mercury, nickel, zinc
Tanning of hides and skins	NMVOC^a^	Chromium
Food and beverage sector	NMVOC^a^, arsenic, cadmium, chromium, copper, mercury, nickel, dioxins + furans, PM_10_^c^, TSP^d^	Chromium, copper, mercury, nickel, lead, zinc, naphthalene, PAHs^b^, toluene, fluoranthene, benzo(g,h,i)perylene, benzo(a)pyrene, benzo(b)fluoranthene
Surface treatment using organic solvents	NMVOC^a^, chromium, copper, nickel, lead, zinc, dichloromethane, naphthalene, PAHs^b^, PM_10_^c^, TSP^d^	Arsenic, cadmium, chromium, copper, mercury, nickel, lead, zinc, 1,2-dichloroethane, trichloroethylene, trichloromethane, organotin compounds, toluene, naphthalene, PAHs^b^
Production of carbon or electro-graphite	NMVOC^a^, PAHs^b^, PM_10_^c^, TSP^d^	

^a^Non-methane volatile organic compounds

^b^Polycyclic aromatic hydrocarbons

^c^Particulate matter

^d^Total suspended particulate matter

### First methodological approach: “*Near vs. far*” analyses

As a first example of this methodology, the odds ratios (ORs) and their 95% confidence intervals (95% CIs) of the several childhood cancers studied in our papers in relation to the analysis of industrial and urban areas as a whole (analysis 1.a), for industrial distances between 2 and 5 km, are shown in Table 3. Statistically significant excess risks were found in children close to:

**Table 3:** ORs of childhood tumors in the proximity of industrial and urban areas

Industrial distance (km)	Tumor	Industrial area	Urban area	Both
		OR (95% CI)	OR (95% CI)	OR (95% CI)
≤ 5	Leukemias [8]	1.13 (0.83–1.56)	1.41 (0.76–2.61)	1.05 (0.77–1.44)
	Renal [5]	1.85 (1.07–3.18)	1.28 (0.35–4.75)	1.90 (1.00–3.59)
	Neuroblastoma [7]	1.30 (0.89–1.91)	1.83 (0.78–4.33)	1.41 (0.90–2.20)
	Bone [4]	NA^a^	NA^a^	NA^a^
	Retinoblastoma [6]	1.33 (0.73–2.44)	2.08 (0.58–7.42)	0.94 (0.45–1.94)
	Hepatic [6]	0.60 (0.24–1.48)	0.31 (0.06–1.75)	0.45 (0.14–1.48)
	Soft tissue sarcomas [6]	0.60 (0.39–0.93)	0 (0–inf)	0.81 (0.48–1.37)
	Germ cell tumors [6]	1.28 (0.68–2.40)	1.76 (0.33–9.52)	1.31 (0.62–2.76)
	Other epithelial neoplasms/melanomas [6]	1.24 (0.29–5.35)	0 (0–inf)	0.58 (0.09–3.63)
	Central nervous system [10]	NA^a^	NA^a^	NA^a^
≤ 4	Leukemias [8]	1.26 (0.94–1.70)	1.66 (1.08–2.55)	1.12 (0.83–1.50)
	Renal [5]	1.91 (1.11–3.29)	1.65 (0.71–3.81)	1.92 (1.00–3.71)
	Neuroblastoma [7]	1.29 (0.88–1.90)	1.46 (0.82–2.60)	1.42 (0.90–2.25)
	Bone [4]	NA^a^	NA^a^	NA^a^
	Retinoblastoma [6]	1.31 (0.71–2.40)	1.16 (0.46–2.92)	0.96 (0.45–2.04)
	Hepatic [6]	0.60 (0.24–1.48)	0.31 (0.06–1.75)	0.45 (0.14–1.48)
	Soft tissue sarcomas [6]	0.59 (0.38–0.93)	0.51 (0.23–1.17)	0.81 (0.47–1.39)
	Germ cell tumors [6]	1.24 (0.65–2.34)	0.98 (0.32–3.02)	1.43 (0.67–3.08)
	Other epithelial neoplasms/melanomas [6]	1.40 (0.32–6.14)	0 (0–inf)	0.73 (0.12–4.62)
	Central nervous system [10]	NA^a^	NA^a^	NA^a^
≤ 3	Leukemias [8]	1.26 (0.97–1.63)	1.29 (0.92–1.80)	1.10 (0.85–1.42)
	Renal [5]	1.96 (1.13–3.39)	1.12 (0.52–2.43)	2.62 (1.34–5.12)
	Neuroblastoma [7]	1.28 (0.87–1.89)	1.38 (0.84–2.27)	1.48 (0.91–2.41)
	Bone [4]	2.33 (1.17–4.63)	4.43 (1.80–10.92)	3.66 (1.53–8.75)
	Retinoblastoma [6]	1.31 (0.71–2.42)	1.33 (0.60–2.93)	0.76 (0.32–1.76)
	Hepatic [6]	0.60 (0.24–1.50)	0.44 (0.12–1.66)	0.36 (0.10–1.42)
	Soft tissue sarcomas [6]	0.64 (0.41–1.00)	0.60 (0.31–1.15)	0.86 (0.48–1.54)
	Germ cell tumors [6]	1.23 (0.64–2.34)	1.28 (0.55–3.00)	1.38 (0.60–3.18)
	Other epithelial neoplasms/melanomas [6]	1.45 (0.33–6.37)	0 (0–inf)	1.08 (0.17–6.93)
	Central nervous system [10]	NA^a^	NA^a^	NA^a^
≤ 2.5	Leukemias [8]	1.31 (1.03–1.67)	1.36 (1.02–1.80)	1.07 (0.84–1.36)
	Renal [5]	1.97 (1.13–3.42)	1.38 (0.67–2.81)	2.62 (1.30–5.30)
	Neuroblastoma [7]	1.33 (0.90–1.97)	1.32 (0.81–2.13)	1.62 (0.98–2.69)
	Bone [4]	2.19 (1.10–4.39)	4.08 (1.72–9.64)	3.89 (1.55–9.76)
	Retinoblastoma [6]	NA^a^	NA^a^	NA^a^
	Hepatic [6]	NA^a^	NA^a^	NA^a^
	Soft tissue sarcomas [6]	NA^a^	NA^a^	NA^a^
	Germ cell tumors [6]	NA^a^	NA^a^	NA^a^
	Other epithelial neoplasms/melanomas [6]	NA^a^	NA^a^	NA^a^
	Central nervous system [10]	NA^a^	NA^a^	NA^a^
≤ 2	Leukemias [8]	1.31 (1.04–1.65)	1.28 (1.00–1.53)	1.00 (0.79–1.26)
	Renal [5]	2.02 (1.16–3.52)	1.37 (0.69–2.73)	3.14 (1.50–6.58)
	Neuroblastoma [7]	1.27 (0.85–1.90)	1.22 (0.76–1.96)	2.12 (1.18–3.83)
	Bone [4]	1.97 (0.97–4.02)	4.02 (1.73–9.34)	3.90 (1.48–10.29)
	Retinoblastoma [6]	1.38 (0.73–2.59)	1.19 (0.57–2.52)	0.69 (0.26–1.84)
	Hepatic [6]	0.65 (0.25–1.68)	0.33 (0.10–1.16)	0.60 (0.13–2.71)
	Soft tissue sarcomas [6]	0.62 (0.39–0.99)	0.62 (0.35–1.11)	1.02 (0.52–1.96)
	Germ cell tumors [6]	1.15 (0.59–2.26)	1.23 (0.56–2.71)	1.62 (0.61–4.32)
	Other epithelial neoplasms/melanomas [6]	1.82 (0.39–8.44)	0.44 (0.05–3.81)	1.06 (0.12–9.09)
	Central nervous system [10]	0.96 (0.73–1.26)	0.90 (0.65–1.24)	1.20 (0.82–1.77)

*OR* odds ratio, *95% CI* 95% confidence interval

^a^Not applicable

- industrial facilities for leukemias (OR 1.31; 95% CI 1.04–1.65 at ≤ 2 km, and OR 1.31; 95% CI 1.03–1.67 at ≤ 2.5 km) and renal cancer [with ORs ranged between 1.85 (95% CI 1.07–3.18) at ≤ 5 km and 2.02 (95% CI 1.07–3.18) at ≤ 2 km];
- urban areas for leukemias (OR 1.28; 95% CI 1.00–1.53 at ≤ 2 km, OR 1.36; 95% CI 1.02–1.80 at ≤ 2.5 km, and OR 1.66; 95% CI 1.08–2.55 at ≤ 4 km) and bone tumors [with ORs ranged between 4.02 (95% CI 1.73–9.34) at ≤ 2 km and 4.43 (95% CI 1.80–10.92) at ≤ 3 km]; and,
- intersection area between industrial and urban sites for renal cancer (with ORs ranged between 1.90 (95% CI 1.00–3.59) at ≤ 5 km and 3.14 (95% CI 1.50–6.58) at ≤ 2 km), neuroblastoma (OR 2.12; 95% CI 1.18–3.83 at ≤ 2 km), and bone tumors [with ORs ranged between 3.66 (95% CI 1.53–8.75) at ≤ 3 km and 3.90 (95% CI 1.48–10.29) at ≤ 2 km].

The ORs of those childhood cancers with statistically significant results and a number of controls and cases ≥ 5, for the “*near vs. far*” analysis by category of industrial group (analysis 1.b) and an industrial distance of ≤ 2.5 km, are shown in Table 4. The following positive associations between certain cancers and residential proximity to specific industrial groups were found:

**Table 4:** ORs of those childhood tumors with significant results for the “*near vs. far*” analysis by category of industrial group (≤ 2.5 km)

Tumor	Industrial group (≤ 2.5 km)	OR (95% CI)
Leukemias [8]	Production and processing of metals	1.69 (1.22–2.34)
	Galvanization	1.86 (1.07–3.21)
	Surface treatment of metals and plastic	1.62 (1.22–2.15)
	Glass and mineral fibers	2.42 (1.49–3.92)
	Pharmaceutical products	1.53 (1.00–2.34)
	Hazardous waste	1.55 (1.06–2.28)
	Surface treatment using organic solvents	1.87 (1.24–2.83)
Renal [5]	Production and processing of metals	1.98 (1.03–3.82)
	Galvanization	2.66 (1.14–6.22)
	Surface treatment of metals and plastic	2.25 (1.24–4.08)
	Glass and mineral fibers	2.69 (1.19–6.08)
	Ceramic	2.35 (1.06–5.21)
	Organic chemical industry	2.22 (1.15–4.26)
	Hazardous waste	2.59 (1.25–5.37)
	Urban waste-water treatment plants	2.14 (1.07–4.30)
	Food and beverage sector	2.19 (1.18–4.07)
Neuroblastoma [7]	Mining	4.67 (1.70–12.81)
Bone [4]	Production and processing of metals	3.30 (1.41–7.77)
	Surface treatment of metals and plastic	2.59 (1.22–5.50)
	Cement and lime	3.89 (1.19–12.77)
	Organic chemical industry	3.07 (1.23–7.62)
	Pharmaceutical products	2.50 (1.01–6.18)
	Urban waste-water treatment plants	2.61 (1.04–6.54)

*OR* odds ratio, *95% CI* 95% confidence interval

- ‘Production and processing of metals, ‘Galvanization’, ‘Surface treatment of metals and plastic’, ‘Glass and mineral fibers’, and ‘Hazardous waste’ ⇔ leukemias and renal tumors;
- ‘Organic chemical industry’ and ‘Urban waste-water treatment plants’ ⇔ renal and bone tumors;
- ‘Pharmaceutical products’ ⇔ leukemias and bone tumors;
- ‘Surface treatment using organic solvents’ ⇔ leukemias;
- ‘Ceramic’ and ‘food and beverage sector’ ⇔ renal tumors;
- ‘Mining’ ⇔ neuroblastoma; and,
- ‘Cement and lime’ ⇔ bone tumors.

As an example of the “*near vs. far*” analysis by category of pollutants (carcinogens and toxic substances) (analysis 1.c) for an industrial distance of ≤ 2.5 km, the ORs of leukemias, and renal and bone tumors are shown in Table 5. Statistically significant excess risks of leukemias and bone tumors were found in the environs of facilities releasing substances included in all IARC groups. In the case of bone tumors, the excess risk was only observed near industries releasing Group 1-carcinogens. According to the categorization of ‘Groups of toxic substances’, statistically significant ORs of leukemias, and renal and bone tumors were found in all groups of toxic substances (with the exception of plasticizers for renal tumors, and volatile organic compounds for bone tumors).

**Table 5:** ORs of childhood tumors for the “*near vs. far*” analysis by category of pollutants (≤ 2.5 km)

Groups of pollutants (≤ 2.5 km)	Leukemias [8]	Renal [5]	Bone [4]
	OR (95% CI)	OR (95% CI)	OR (95% CI)
*IARC groups* ^*a*^			
Group 1	1.35 (1.05–1.73)	2.02 (1.15–3.52)	2.28 (1.13–4.62)
Group 2A	1.48 (1.13–1.93)	2.13 (1.19–3.81)	1.97 (0.92–4.22)
Group 2B	1.54 (1.14–2.07)	2.26 (1.22–4.19)	2.20 (0.95–5.07)
*Groups of toxic substances* ^*b*^			
Metals	1.40 (1.08–1.80)	2.05 (1.16–3.63)	2.25 (1.09–4.63)
Pesticides	1.61 (1.14–2.27)	2.88 (1.46–5.65)	3.43 (1.42–8.28)
PACs	1.57 (1.16–2.11)	2.16 (1.16–4.03)	2.50 (1.08–5.80)
Non-HPCs	1.71 (1.21–2.40)	2.18 (1.07–4.45)	3.06 (1.11–8.46)
Plasticizers	1.67 (1.10–2.55)	1.32 (0.53–3.29)	3.46 (1.06–11.27)
POPs	1.58 (1.19–2.10)	2.51 (1.38–4.56)	2.86 (1.31–6.26)
VOCs	1.38 (1.07–1.78)	1.90 (1.08–3.35)	1.98 (0.96–4.09)
Solvents	1.61 (1.20–2.14)	2.37 (1.30–4.34)	2.30 (1.04–5.09)
Other	1.37 (1.06–1.77)	2.04 (1.16–3.59)	2.40 (1.18–4.90)

*OR* odds ratio, *95% CI* 95% confidence interval

^a^IARC carcinogenic classification: Group 1: carcinogens to humans (arsenic and compounds, cadmium and compounds, chromium and compounds, nickel and compounds, lindane, dioxins + furans, polychlorinated biphenyls, trichloroethylene, vinyl chloride, benzene, ethylene oxide, polycyclic aromatic hydrocarbons, particulate matter (PM_10_), total suspended particulate matter, and benzo(a)pyrene); Group 2A: probably carcinogenic to humans (lead and compounds, dichloromethane, tetrachloroethylene, DDT, and hexabromobiphenyl); Group 2B: possibly carcinogenic to humans (chlordane, 1,2-dichloroethane, heptachlor, hexachlorobenzene, 1,2,3,4,5,6-hexachlorocyclohexane, mirex, pentachlorophenol, tetrachloromethane, trichloromethane, ethyl benzene, naphthalene, di-(2-ethyl hexyl) phthalate, cobalt and compounds, benzo(b)fluoranthene, benzo(k)fluoranthene, and indeno(1,2,3-cd)pyrene)

^b^Metals (arsenic and compounds, cadmium and compounds, chromium and compounds, copper and compounds, mercury and compounds, nickel and compounds, lead and compounds, zinc and compounds, thallium, antimony, cobalt, manganese, and vanadium); Pesticides (alachlor, aldrin, atrazine, chlordane, chlorfenvinphos, chlorpyrifos, DDT, dieldrin, diuron, endosulfan, endrin, heptachlor, lindane, mirex, pentachlorobenzene, pentachlorophenol, simazine, isoproturon, organotin compounds, tributyltin and compounds, triphenyltin and compounds, trifluralin, and isodrin); PACs: Polycyclic aromatic chemicals (anthracene, polycyclic aromatic hydrocarbons, fluoranthene, benzo(g,h,i)perylene, benzo(a)pyrene, benzo(b)fluoranthene, benzo(k)fluoranthene, and indeno(1,2,3-cd)pyrene); Non-HPCs: Non-halogenated phenolic chemicals (nonylphenol and nonylphenol ethoxylates, and octylphenols and octylphenol ethoxylates); Plasticizers (di-(2-ethyl hexyl) phthalate); POPs: Persistent organic pollutants (aldrin, chlordane, DDT, dieldrin, endosulfan, endrin, heptachlor, hexachlorobenzene, 1,2,3,4,5,6-hexachlorocyclohexane, lindane, mirex, dioxins + furans, pentachlorobenzene, polychlorinated biphenyls, brominated diphenylethers, organotin compounds, polycyclic aromatic hydrocarbons, hexabromobiphenyl, benzo(a)pyrene, benzo(b)fluoranthene, and benzo(k)fluoranthene); VOCs: Volatile organic compounds (non-methane volatile organic compounds, 1,2-dichloroethane, dichloromethane, hexachlorobutadiene, tetrachloroethylene, trichlorobenzenes, 1,1,1-trichloroethane, trichloroethylene, trichloromethane, vinyl chloride, benzene, ethyl benzene, ethylene oxide, and naphthalene); Solvents (1,2-dichloroethane, dichloromethane, tetrachloroethylene, trichlorobenzenes, 1,1,1-trichloroethane, trichloroethylene, trichloromethane, benzene, ethyl benzene, toluene, and xylenes); Other (tetrachloromethane, particulate matter (PM_10_), and total suspended particulate matter)

Finally, the ORs of those childhood cancers with significant results and a number of controls and cases ≥ 5, for the “*near vs. far*” analysis by specific pollutant (analysis 1.d) and an industrial distance of ≤ 2.5 km, are shown in Table 6. The highest ORs were found in the environs of industries releasing:

**Table 6:** ORs of those childhood tumors with significant results for the “*near vs. far*” analysis by specific carcinogen (≤ 2.5 km)

Pollutant (≤ 2.5 km)	IARC group	Leukemias [8]	Renal [5]
		OR (95% CI)	OR (95% CI)
Arsenic and compounds	1	1.50 (1.14–1.98)	1.88 (0.85–4.16)
Benzene	1	1.50 (1.05–2.13)	3.15 (1.27–7.81)
Benzo(a)pyrene	1	2.59 (1.09–6.16)	0 (0–inf)
Cadmium and compounds	1	1.50 (1.14–1.98)	2.20 (1.02–4.76)
Chromium and compounds	1	1.49 (1.15–1.93)	2.57 (1.23–5.37)
Nickel and compounds	1	1.46 (1.12–1.89)	2.43 (1.17–5.06)
Particulate matter (PM_10_)	1	1.33 (1.02–1.73)	2.51 (1.21–5.20)
PCDD + PCDF (dioxins + furans)	1	1.58 (1.16–2.16)	3.33 (1.42–7.82)
Polychlorinated biphenyls	1	1.30 (0.83–2.04)	3.60 (1.10–11.76)
Polycyclic aromatic hydrocarbons	1	1.54 (1.14–2.08)	2.69 (1.16–6.24)
Total suspended particulate matter	1	1.36 (0.99–1.83)	2.56 (1.17–5.57)
Lead and compounds	2A	1.48 (1.13–1.94)	1.87 (0.85–4.12)
Tetrachloroethylene	2A	1.65 (1.13–2.41)	3.48 (1.30–9.31)
1,2-Dichloroethane	2B	1.34 (0.89–2.01)	4.24 (1.66–10.85)
Cobalt and compounds	2B	1.02 (0.60–1.73)	3.73 (1.28–10.85)
Di-(2-ethyl hexyl) phthalate	2B	1.67 (1.10–2.55)	3.46 (1.06–11.27)
Dichloromethane	2B	1.65 (1.11–2.45)	2.92 (1.14–7.45)
Indeno(1,2,3-cd)pyrene	2B	2.59 (1.09–6.16)	0 (0–inf)
Naphthalene	2B	1.48 (1.00–2.17)	2.79 (1.01–7.67)
Tetrachloromethane	2B	2.23 (1.35–3.68)	3.30 (1.06–10.29)
Trichloromethane	2B	1.11 (0.71–1.74)	3.30 (1.06–10.29)

*OR* odds ratio, *95% CI* 95% confidence interval

- ‘Benzo(a)pyrene’ (OR 2.59; 95% CI 1.06–6.16), ‘Indeno(1,2,3-cd)pyrene’ (OR 2.59; 95% CI 1.06–6.16), and ‘Tetrachloromethane’ (OR 2.23; 95% CI 1.35–3.68), for leukemias; and,
- ‘1,2-Dichloromethane’ (OR 4.24; 95% CI 1.66–10.85), ‘Cobalt and compounds’ (OR 3.73; 95% CI 1.28–10.85), and ‘Polychlorinated biphenyls’ (OR 3.60; 95% CI 1.10–11.76), for renal tumors.

### Second methodological approach: “*Risk gradient*” analyses

As an example of this methodology applied to renal tumors, statistically significant radial effects (rise in OR with increasing proximity to industries, according to concentric rings) in the vicinity of industrial installations, both overall (analysis 2.a) and by industrial group (analysis 2.b), were detected (see Table 7) in all industries as a whole (*p*-trend = 0.007), and in the following industrial groups: ‘Surface treatment of metals and plastic’ (*p*-trend = 0.012), ‘Urban and waste-water treatment plants’ (*p*-trend = 0.034), ‘Food and beverage sector’ (*p*-trend = 0.040), and ‘Glass and mineral fibers’ (*p*-trend = 0.046).

**Table 7:** ORs of childhood renal tumors for the “*risk gradient*” analyses with significant radial effects

Analysis	Analysis with categorical “*exposure*” variable (reference: [5–50 km])					Analysis with continuous “*exposure*” variable	
	[0–1 km)	[1–2 km)	[2–3 km)	[3–4 km)	[4–5 km]	OR	*p*-trend
	OR (95% CI)	OR (95% CI)	OR (95% CI)	OR (95% CI)	OR (95% CI)		
*All industries as a whole (analysis 2.a)*	2.07 (1.13–3.78)	1.96 (1.09–3.54)	1.74 (0.90–3.35)	1.53 (0.65–3.58)	0.99 (0.35–2.86)	1.16	0.007
*Category of industrial group (analysis 2.b)*							
Surface treatment of metals and plastic	2.60 (1.27–5.33)	1.92 (0.98–3.77)	1.39 (0.70–2.76)	1.53 (0.73–3.21)	1.60 (0.66–3.88)	1.18	0.012
Glass and mineral fibers	3.43 (0.54–21.76)	5.28 (1.63–17.12)	1.19 (0.39–3.60)	1.69 (0.56–5.11)	1.93 (0.67–5.56)	1.28	0.046
Urban waste-water treatment plants	2.35 (0.57–9.69)	2.23 (0.95–5.28)	1.89 (0.88–4.07)	2.35 (1.15–4.78)	1.46 (0.67–3.18)	1.19	0.034
Food and beverage sector	2.45 (1.04–5.80)	1.86 (0.93–3.72)	1.62 (0.72–3.69)	1.11 (0.43–2.88)	1.65 (0.78–3.48)	1.15	0.040

*OR* odds ratio, *95% CI* 95% confidence interval

## Discussion

In the present paper, two different methodological approaches to perform the statistical analyses in the study of risk of childhood cancer in the vicinity of industrial and urban sites have been used by our group. These two approaches are complementary, none is preferable to the other: the “*near vs. far*” approach is often used as a first step in the study of cancer risk in the environs of pollution sources, whereas the second approach (“*risk gradient*” analysis) is often used to complement the results obtained in the first approach, giving a more detailed information about the behavior of the risk in different partitions of the “near” zone. Positive results or positive associations found in both approaches support and reinforce the hypothesis of a “real” excess risk in the vicinity of the pollution sources analyzed in the study. However, the main limitation of these methodological approaches is the choice of the radius in the “*near vs. far*” analysis and the critical categorization in concentric rings in the “*risk gradient*” analysis, although our industrial distances are in line with the distances used by other authors [13–15]. Another limitation is the assumption of the linear trend in the risk in the “*risk gradient*” analysis, something that might not be true.

In relation to alternative approaches published by other authors, Barbone et al. [16] used an alternative strategy in the definition of “exposure” variable for the “*near vs. far*” analyses, based on deciles of the distribution of the industrial and urban distances, in a case–control study of air pollution and lung cancer in Trieste (Italy). In that study, there were one urban nucleus and three industrial pollution sources: a shipyard, an iron foundry, and an incinerator. Our group adapted their strategy in a similar case–control study of lung cancer risk and pollution in Asturias (Spain) [17, 18], with 48 industrial facilities, and 4 urban nuclei with numbers of inhabitants ranged between 24,735 and 263,547 inhabitants. However, when the sizes of the towns differ considerably among them, that methodology causes an irregular distribution of cases and controls between the zones around the towns, since all towns have the same radius for the “urban area” and only a few big cities include the majority of cases and controls. Because of this, we consider that our methodology is more appropriate for analyses with many towns and very different size of the towns (see Fig. 2).

**Fig. 1:**
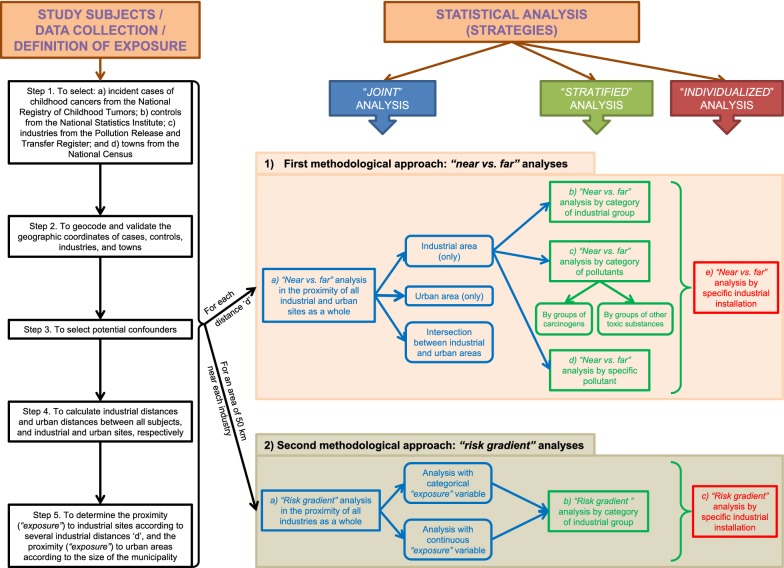
Diagram of the case–control study about the association between proximity to industrial and urban areas and childhood cancer risk

The methodology used in the present paper can be extrapolated to other tumors (even in the general population) and/or other countries with a National Registry of Cancer. In fact, the methodology has already been implemented in the ‘MCC-Pollution’ study (included in the ‘MCC-Spain’ project [19]), a population-based multicase–control study that analyzes the risk cancer in tumors of high incidence in the Spanish general population associated with residential proximity to industrial facilities [20]. The diagram of Fig. 1 can also be generalized to other chronic diseases which could be related to environmental risk factors. In general, our results suggest possible associations between residential proximity to specific industrial and urban zones and risk of some childhood cancers, especially leukemias, neuroblastoma, and renal and bone tumors. In relation to industrial sites, this risk was found in children living in the environs of several industrial types and industries releasing specific carcinogens and toxic substances.

This methodology can be applied directly to other hazardous point sources and toxic hotspots, such as e-waste recycling sites and illegal hazardous dumps [21], and it can also be easily adapted when the pollution focus is not a single point (e.g.: industry, urban nucleus) but a line (e.g.: road traffic, motorway, polluted river) [12] or a polygon (e.g.: crops treated with pesticides) [9]. Taking the dispersion of air pollutants into account, the methodology allows the possibility of using information about wind roses (which include the direction and speed of prevailing winds around specific monitoring points) together with the distance to refine the definition of industrial proximity to pollution sources [17].

To replicate this methodology in other countries, in relation to the location of subjects (cases and controls) and pollution sources (industries and towns), the children’s domiciles (and geographic coordinates) for cases and controls should be provided by the respective National Registry of Childhood Tumors and National Statistics Institute (see Fig. 1), under collaboration agreements, because they are usually very sensitive data (see *Availability of data and material* section). In the case of the industries, all information about industrial plants, including geographic coordinates is publicly available. In the case of the towns, the geographic coordinates of towns’ centroids are publicly available in the Spanish Census. On the other hand, the tools used in the geocoding strategies for all these elements (cases, controls, industries, and towns) are open access (see *Methods* section). The methodology used in the paper requires the compulsory use of geographic coordinates to be applied correctly in the different analyses.

Epidemiological studies of childhood cancer in relation to proximity to pollution foci have reached great importance recently [22–27], and industrial registers of toxic substances as the E-PRTR provide a tool for the monitoring and surveillance of harmful effects of these industrial pollutants, some of them carcinogenic, on the human health. In this sense, our experience is being positive because our study is providing some epidemiological clues that residing in the vicinity of certain industrial and urban areas may be a risk factor for some types of childhood cancers.

With regard to childhood leukemias and the pollution sources analyzed in our previous papers, our findings about proximity to industrial groups (see Table 4) are consistent with other studies in relation to the excess risk found in the environs of the metal industry (which includes ‘Production and processing of metals’, ‘Galvanization’, and ‘Surface treatment of metals and plastic’) [28, 29] and installations for the manufacture of ‘Glass and mineral fibers’ [28], although other authors did not find associations with proximity to incinerators (‘Hazardous waste’) [15]. In relation to specific carcinogens and groups of pollutants, some authors found a possible increased risk of some types of childhood leukemias in children living within 3 km of industrial dichloromethane releases (OR 1.64; 95% CI 1.15–2.32) [30], very similar to our results for this pollutant at 2.5 km (OR 1.65; 95% CI 1.11–2.45). Other authors have also found associations between benzene exposure and childhood risk of acute lymphocytic leukemia [31–33], in line with our results (see Table 6). Finally, our findings about proximity to urban areas (see Table 3), as a proxy of urban pollution, are consistent with other papers [12, 34, 35].

With respect to proximity to environmental exposures and childhood renal tumors, the few studies focused on residential proximity to environmental pollution sources did not find associations in relation to hazardous waste sites [36] or major roadways [27]. However, some authors have found associations between children prenatally exposed to polycyclic aromatic hydrocarbons during the third trimester and risk of Wilms’s tumor (the main histologic type of childhood renal tumors) [37], something that could be related to our findings about this type of pollutant (see Table 6).

Insofar as neuroblastoma and environmental exposures are concerned, Heck et al. [38] did not find associations between exposure to traffic pollution and neuroblastoma. In our study about this cancer, the excess risks found in the urban areas were not statistically significant (see Table 3). However, the same authors found increased risks of neuroblastoma with regard to a higher maternal exposure to chromium and polycyclic aromatic hydrocarbons in a radius of 2.5 km, very similar to the non-statistically excess risks found in our study (data not shown).

In relation to childhood bone tumors and proximity to industrial areas, there are few studies focused on this aspect. Pan et al. [39] found a higher mortality of bone tumors in the environs of petrochemical industries, whereas Wulff et al. [40] found an excess risk of bone cancer near a smelter. Our results about ‘Organic chemical industry’ and ‘Production and processing of metals’ yielded high excess risks (see Table 4). With respect to childhood bone tumors and proximity to urban areas, the majority of the studies existing in the literature found significant excess risks in children living in urban zones [41–43], in line with our findings (see Table 3). However, other authors did not find associations between proximity to urban zones and risk of childhood bone cancer [44].

As future perspectives, research is still needed on air pollution, especially in industrial and urban zones, and childhood cancer to guide policies for the reduction of emission of toxic and carcinogenic substances and protection of public health. Direct epidemiologic observation of exposed children for evaluating the magnitude of air pollution and large-scale epidemiologic studies of environmental exposures and childhood cancer are needed [45]. Moreover, surveillance systems for residential and occupational exposures, and clusters of childhood cancers should be implemented to prevent childhood cancer risk [46]. Finally, identification and control of environmental risk factors that may cause cancer in children is the single most effective strategy for cancer prevention [23]. As Nelson et al. [47] say, reducing environmental hazards associated with residential exposures could substantially reduce the human burden of childhood cancer and result in significant annual and lifetime savings.

## Conclusions

The methodological approaches used by our group have proved to be very useful and flexible tools to analyze the excess risk of childhood cancers in the vicinity of industrial and urban areas, which can be extrapolated and generalized to other cancers and chronic diseases, and adapted to other types of pollution sources.

## Methods

A population-based case–control study of incident childhood cancers in Spain and their relationship with residential proximity to environmental pollution sources, in this case, industrial and urban areas, was designed. The diagram of our study is shown in Fig. 1: the first part depicts the several steps about the study subjects, data collection, and definition of the exposure, whereas the second part represents the strategies of statistical analysis used in our papers [4–8, 10].

### Study subjects/data collection/definition of exposure

*Step 1* Cases, controls, industries, and towns were selected as follows:

(A) Cases: in our case, incident cases of childhood cancers (0–14 years) were gathered from the Spanish Registry of Childhood Tumors, for Autonomous Regions with 100% coverage between 1996 and 2011: (a) Leukemias, myeloproliferative diseases, and myelodysplastic diseases [code I, according to the International Classification of Diseases for Oncology, 3rd revision (ICCC-3)]; (b) Renal tumors (code VI, ICCC-3); (c) Neuroblastoma and other peripheral nervous cell tumors (code IV, ICCC-3); (d) Malignant bone tumors (code VIII, ICCC-3); (e) Retinoblastoma (code V, ICCC-3); (f) Hepatic tumors (code VII, ICCC-3); (g) Soft tissue and other extraosseous sarcomas (code IX, ICCC-3); (h) Germ cell tumors, trophoblastic tumors, and neoplasms of gonads (code X, ICCC-3); (i) Other malignant epithelial neoplasms and malignant melanomas (code XI, ICCC-3); and, (j) Central nervous system and miscellaneous intracranial and intraspinal neoplasms (code III, ICCC-3) [48].
(B) Controls: from among all single live births registered in the Spanish National Statistics Institute [49] for the study period, six controls per case were chosen by simple random sampling, individually matched to cases by autonomous region of residence, sex, and year of birth.
(C) Industries: data on industries were provided from the E-PRTR [50] through the Spanish Ministry for the Ecological Transition [51], for the year 2009.
(D) Towns: urban locations (towns ≥ 75,000 inhabitants, according to the 2001 Spanish Census [52]) were used.

*Step 2* The geographic coordinates of cases, controls, industries, and towns were geocoded and validated, as follows:

(A) Geocoding strategy for cases and controls: each child’s last domicile was geocoded using Google Maps JavaScript V3 [53]. The obtained latitude and longitude coordinates were projected into ETRS89/Universal Transverse Mercator (UTM) zone 30N (EPSG:25830) coordinates using QGIS software [54], and subsequently converted into ED50/UTM zone 30 (EPSG:23030) coordinates using the R software [55]. After this, the coordinates were validated and those where the addresses and the coordinates matched were chosen. For this validation process, the inverse method was applied, getting the home addresses of the obtained coordinates and comparing these new addresses (street number and name, postal code, and city/town name) to the original addresses. Lastly, in the final ED50/UTM zone 30 coordinates of the children’s domiciles, the last digit of the pair of coordinates (X, Y) was assigned randomly with the purpose of preserving their confidentiality. With respect to the cases, 87% of their domiciles were successfully validated. The remaining 13% of cases were fairly uniformly distributed through the different autonomous regions and, therefore, we declared that our data were not biased in this sense. In relation to the controls, initially, only 2% of their addresses could not validate. Owing to this small number of failures in the coordinates, we decided to select more controls to replace this small percentage and, finally, we geocoded and validated this last group to end up with six controls with valid coordinates for each case.
(B) Geocoding strategy for industries: the original geographic location of each industrial facility included in the E-PRTR (longitude/latitude projection) was converted into ED50/UTM zone 30 coordinates using the R software [55], and subsequently validated following the methodology used for our group in the validation of the EPER [56], the industrial register to which the E-PRTR replaced in 2007. However, owing to the presence of errors in many of the industrial locations, every single address was thoroughly checked to ensure that the location of the industrial plant was exactly where it should be. The following tools were used: (1) the Spanish Agricultural Plot Geographic Information System (SIGPAC) Viewer [which includes topographic maps showing the names of industrial plants, and orthophotos (digitalized aerial images)] [57]; (2) Google Earth (with the street-view application); (3) the “Yellow pages” web page (which allows for a search of companies and addresses) [58]; (4) the Google Maps server [59]; and (5) the web pages of the industrial companies.
(C) Geocoding strategy for towns: municipal centroids (not polygonal centroids) of towns in which the children resided were used. In Spain, these municipal centroids are located in the centers of the most populated areas, where the main church and/or the town hall tend to be located. Every single municipal centroid was meticulously checked as in the geocoding strategy for industries, using the Google Maps server [59], Google Earth, and the SIGPAC viewer [57].

*Step 3* Sociodemographic variables for all children as potential confounders were selected. These variables were provided by the 2001 Spanish Census [52] at a census tract level (for their unavailability at an individual level), and included: (a) percentage of illiteracy; (b) percentage of unemployment; and (c) socioeconomic status (based on the occupation of the head of the family): it ranged from 0.46 to 1.57, where the lower value corresponded to the worst socioeconomic status and the higher values to better socioeconomic status.

*Step 4* Euclidean distances between all children and industries (industrial distances) and towns (urban distances) were calculated using the R software [55].

**Fig. 2:**
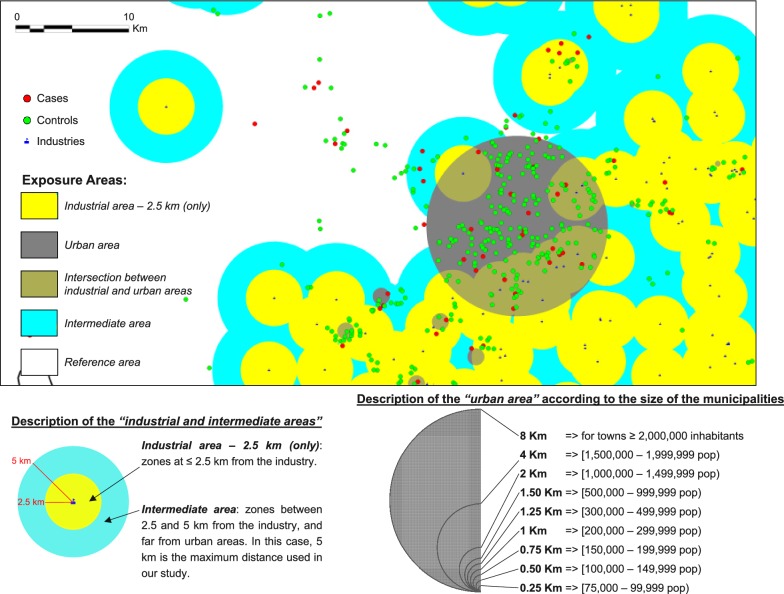
Example of exposure areas to industrial and urban zones, for an industrial distance of 2.5 km

*Step 5* Finally, the “*exposure*” variable (in our case, the proximity to industries, according to several industrial distances ‘d’, and proximity to urban areas, according to the size of the municipality) was determined. Figure 2 shows an example of exposure areas to industrial and urban sites, for an industrial distance of 2.5 km.

### Statistical analysis (strategies)

Two methodological approaches using mixed multiple unconditional logistic regression models to estimate ORs were developed, using the R software [55]. For each one of the two approaches, three strategies of analysis (see Fig. 1) were implemented: (a) “*Joint*” analysis, where the risk of childhood cancer in the vicinity of all industries and towns as a whole was studied; (b) “*Stratified*” analysis, where the excess risk in the environs of industrial areas was stratified, according to: categories of industrial groups (activities) included in the E-PRTR, categories of pollutants (industries releasing groups of known and suspected carcinogens, and other toxic chemical substances), and by specific pollutant; and (c) “*Individualized*” analysis, where the excess risk in the environs of individually selected industrial plants was analyzed.

(1) First methodological approach: “*near vs. far*” analyses.

Potential excess risks of cancers in children living near (“near”) versus those living far (“far”) from industrial and urban areas were assessed, comparing the ratio between the number of cases and controls in zones close to industrial/urban areas and number of cases and controls in zones far from these pollutant sources (OR_near *vs*. far_), and adjusting by potential confounders. Five “*near vs. far*” analyses were performed (see Fig. 1):

- “*Near vs. far*” analysis in the proximity of all industrial and urban sites as a whole, for industrial area (only), urban area (only), and intersection between industrial and urban areas:
  $$\begin{aligned} & \forall c \in C = \left\{ {childhood\;cancers\;studied} \right\}, \;\;\forall d \in D = \left\{ {industrial\; distances} \right\} \\ & logit = \log \left( {\frac{{P\left( {Y = 1} \right)}}{{1 - P\left( {Y = 1} \right)}}} \right) = \beta_{0} + \beta_{1} IndusUrban_{cid} \\&\quad + \mathop \sum \limits_{j = 2}^{n} \beta_{j} MatchConf_{cij} \\ & Y\;is\; the\; case{-}control \;status \;\left( {1 = case, \;0 = control} \right), \\ & i = 1, \ldots , no.\;of\; children \;with\; tumor\; c, \\ & n = no.\;of\; matching\;factors \;and\; other\; potential \;confounders. \\ \end{aligned}$$
  Each subject $$i$$ was classified into one of the following five categories of the “exposure” variable $$(IndusUrban_{cid} )$$ for each tumor $$c$$ and industrial distance $$d$$: (1) residence in the “*industrial area − d km (only)*”, defined in terms of proximity to industrial facilities, on the basis of the industrial distance $$d$$; (2) residence in the “*urban area (only)*”, taking the areas defined by urban distances, according to the size and spatial characteristics of the municipalities in Spain; (3) residence in the “*intersection between industrial and urban areas*”; (4) residence in the “*intermediate area*”, defined as zones forming a “ring” between $$d$$ and $$max\left\{ D \right\}$$ km around the industries; and, (5) residence within the “*reference area*”, consisting of zones with children having no industries within $$max\left\{ D \right\}$$ km of their residences and far from urban areas (see Fig. 2). A total of $$card\left( D \right)$$ independent models were included in this analysis, and all models included matching factors (autonomous region of residence as a random effect, and sex and year of birth as fixed effects) and the potential confounders ($$MatchConf_{cij} )$$ previously mentioned (percentages of illiteracy and unemployment, and socioeconomic status).
- “*Near vs. far*” analysis by category of industrial group, stratifying the excess risk found in industrial areas by categories of industrial groups, according to the E-PRTR (see Table 1). The statistical model is analogous to the previous one. In this case, an exposure variable $(IndusGroup_{cikd} )$ for each tumor $c$ and industrial distance $d$ was created, in which the subject $i$ was classified as resident near the specific “*industrial group*” $k$ (with $k$ = 1, …, no. of industrial groups), if the child resided at ≤ $d$ km from any installation belonging to the industrial group in question, and resident in the reference area, if the child resided at > $max\left\{ D \right\}$ km from any industry and far from urban areas. A total of $dim\left( k \right)$ independent models were included in this analysis, and the remaining variables of the models were the same as in the above analysis.
- “*Near vs. far*” analysis by category of pollutants, stratifying the risk near industrial areas by the following categories of pollutants: (a) Groups of known or suspected carcinogens included in the IARC (Group 1—carcinogens to humans, Group 2A—probably carcinogenic to humans, and Group 2B—possibly carcinogenic to humans); and, (b) Groups of toxic substances created by our groups in previous studies [5, 8]: metals, pesticides, polycyclic aromatic chemicals, non-halogenated phenolic chemicals, plasticizers, persistent organic pollutants, volatile organic compounds, solvents, and other. The statistical model is analogous to the first model. An exposure variable for each tumor $$c$$ and industrial distance $$d$$ ($$SubstanceGroup_{cild} )$$ was created, where each subject $$i$$ was categorized as resident near industries releasing the specific “*group of carcinogenic/toxic substances*” $$l$$ (with $$l$$ = 1, …, no. of groups of carcinogens and toxic substances) or resident in the reference area, analogous to the previous analysis. A total of $$dim\left( l \right)$$ independent models were included in this analysis, and the remaining variables of the models were the same as in the first model.
- “*Near vs. far*” analysis by specific pollutant. The statistical model is analogous to the first model. An exposure variable for each model ($$Pollutant_{cimd} )$$ was created, where each subject $$i$$ was categorized as resident near industries releasing the specific “*pollutant*” $$m$$ (with $$m$$ = 1, …, no. of specific industrial pollutants) or resident in the “*reference area*”, analogous to the previous analyses. A total of $$dim\left( m \right)$$ independent models were included in this analysis, and the remaining variables of the models were the same as in the first model.
- “*Near vs. far*” analysis by specific industrial installation, individually. The statistical model is analogous to the first model. An exposure variable for each model ($$Installation_{cifd} )$$ was created, where each subject $$i$$ was categorized as resident near the specific “*industry*” $$f$$ (with $$f$$ = 1, …, no. of industrial installations) or resident in the reference area, analogous to the previous analyses. The remaining variables were the same as in the first model.

(2) Second methodological approach: “*Risk gradient*” analyses.

To assess the risk gradient in the vicinity of industrial installations (i.e., the rise in OR with increasing proximity to industries, according to concentric rings between 0 km and $max\left\{ D \right\}$ km), three analyses were performed (see Fig. 1). These analyses were confined to an area of $10*max\left\{ D \right\}$ km surrounding each installation, and the ORs were estimated using mixed multiple unconditional logistic regression models.

**Fig. 3:**
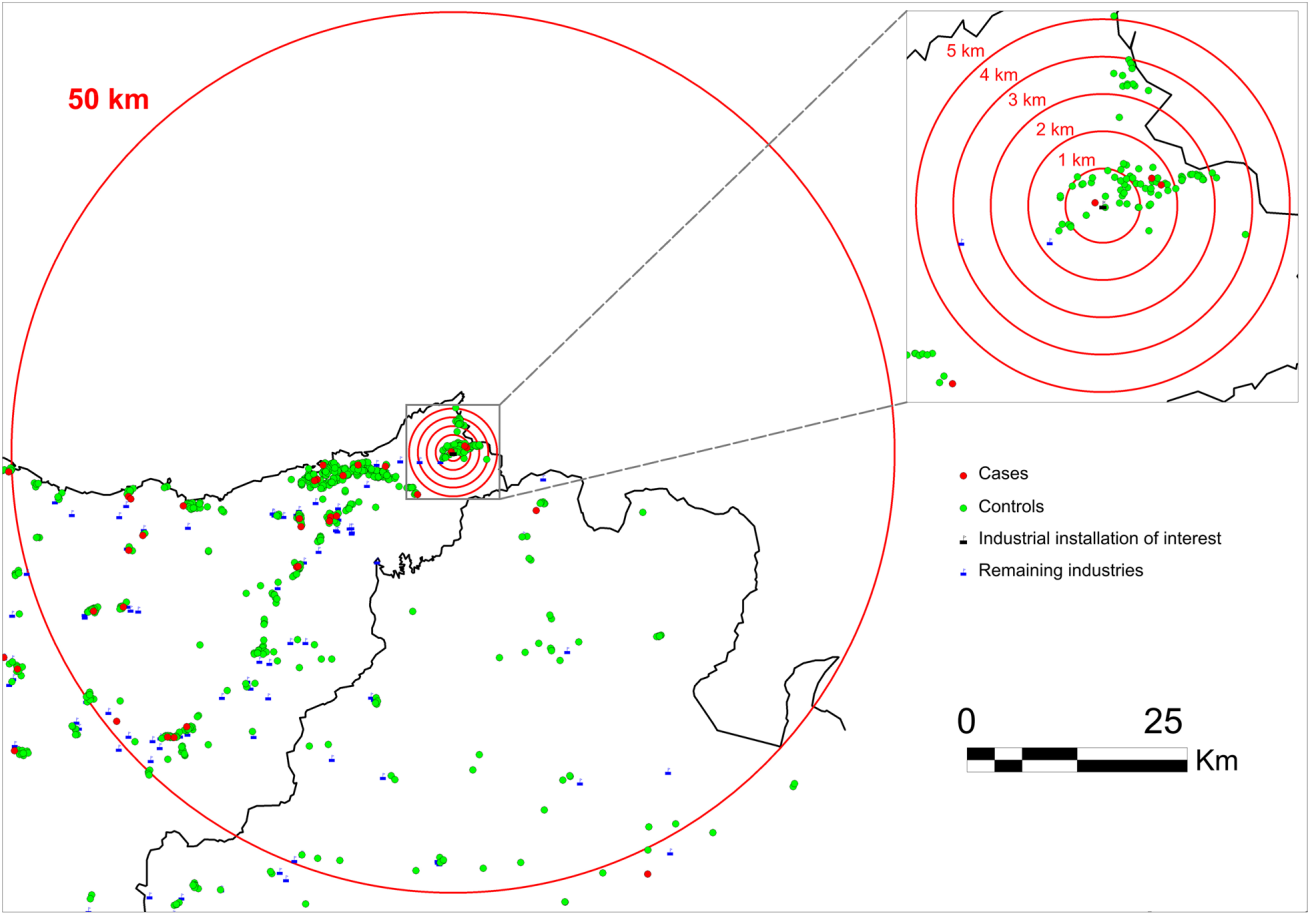
Example of the “*risk gradient*” analysis by specific industrial installation (analysis 2.c)

- “*Risk gradient*” analysis in the proximity of all industries as a whole: for each tumor $c$ and subject $i$, a new variable, “$minimum distance_{ci}$” was calculated as:
  $$\begin{aligned} & {\text{minimum}}\;{\text{distance}}_{\text{ci}} = \hbox{min} \left\{ {{\text{industrial}}\;{\text{distance}}_{\text{cif}} } \right\}_{\text{f}} \\ & i = 1, \ldots , no.\;of \;children \;with\; tumor\; c, \\ & f = 1, \ldots , \;no. \;of\; industrial\; installations, \\ \end{aligned}$$
  where $industrial distance_{cif}$ is the distance between child $i$ and facility $f$ for each tumor $c$. This new explanatory variable was categorized in concentric rings (an example of categorization can be: 0 − $d_{1}$ km, $d_{1}$ − $d_{2}$ km, …, $d_{n - 1}$ − $d_{n}$ km, and reference: $d_{n}$ − $10*max\left\{ D \right\}$ km, being $D = \left\{ {d_{1} , d_{2} , \ldots ,d_{n - 1} ,d_{n} } \right\}$ the set of the industrial distances). This was included in a first model as a categorical variable to estimate the effect of the respective distances, and in a second model as a continuous variable to ascertain the existence of radial effects (rise in OR with increasing proximity to an installation). The likelihood ratio test was applied to compute the statistical significance of such minimum distance-related effects.
- “*Risk gradient*” analysis by category of industrial group: for each tumor $c$, subject $i$, and industrial group $k$, a total of ${ \dim }\left( k \right)$ new variables “$minimum \;distance\_industrial \;group_{cik}$” were calculated as:
  $$\begin{aligned} & {\text{minimum }}\;{\text{distance}}\_{\text{industrial }}\;{\text{group}}_{\text{cik}} = \hbox{min} \left\{ {{\text{industrial }}\;{\text{group}}\;{\text{distance}}_{{ {\text{cip}}}} } \right\}_{\text{p}} \\ & i = 1, \ldots , no.\;of\; children\; with\; tumor\; c, \\ & k = 1, \ldots , no.\; of\; industrial \;groups, \\ & p = 1, \ldots , no.\;of\; facilities \;belonging\; to\; industrial\; group\; k, \\ \end{aligned}$$
  where $industrial\; group \;distance_{cip}$ is the distance between child $i$ and facility $p$ belonging to industrial group $k$, for each tumor $c$. These new explanatory variables were categorized in concentric rings as in the previous analysis. These were included in the models as categorical and continuous variables (in separate models as in the previous analysis), and children that had some industry other than the group analyzed within a radius of $max\left\{ D \right\}$ km of the municipal centroid were excluded.
- “*Risk gradient*” analysis specific industrial installation: for each tumor $c$, subject $i$, and industrial installation $f$, a new variable $industrial\; distance_{cif}$ was calculated as the distance between child $i$ and facility $f$ for each tumor $c$. This new explanatory variable was categorized in concentric rings as in the first analysis and included in the models as both a categorical and a continuous variable (in separate models as in the first “*risk gradient*” analysis). Figure 3 shows an example of this analysis for a specific industrial installation.

## Data Availability

The data are the geographic coordinates of the address of cases and controls. The authors cannot provide these individual coordinates because they are under protection by the Spanish Organic Law 15/1999 on Protection of Personal Data (LOPD). Privacy, confidentiality, and rights of the cases and controls were ensured by changing the last digits of every coordinate (X, Y) by a random number. Data are from the “Industrial pollution and childhood cancer incidence in Spain” study and authors may be contacted at Carlos III Institute of Health (Madrid, Spain): Dr. Rebeca Ramis, rramis@isciii.es.

## Abbreviations

PRTR: Pollutant Release and Transfer Register
E-PRTR: European Pollutant Release and Transfer Register
IARC: International Agency for Research on Cancer
ORs: odds ratios
95% CIs: 95% confidence intervals
ICCC-3: International Classification of Diseases for Oncology, 3rd revision
UTM: Universal Transverse Mercator
SIGPAC: Spanish Agricultural Plot Geographic Information System
